# Mental Hospitals and After-Care

**Published:** 1914-05-16

**Authors:** 


### MENTAL HOSPITALS AND AFTER-CARE.

In recent times many advances have been made in regard to the treatment of the insane; and, even were it granted that the recovery rate has not perceptibly altered (as some critics maintain), the conditions of life during the period of mental illness are certainly more cheerful and more salubrious. Much, too, is being done in the direction of prophylaxis. The due carrying out of the provisions of the Mental Deficiency Act, and the consequent prevention of defectives from laying the burden of their degenerate offspring upon the community; the efforts of the eugenists to place more clearly before the public the disastrous effects of the marriage of the unfit; the more adequate supervision and treatment of syphilis, which the Royal Commission on Venereal Diseases will doubtless advocate; these and other measures give rise to the hope that in the comparatively near future there may be some marked reduction in the numbers of the insane. In addition to prophylaxis and to treatment of insanity there is, however, another aspect of the problem of mental disorder which is not sufficiently kept in view; that is, the care of those who, formerly of unsound mind, have recovered their mental stability. The public which looks almost with terror upon the insane regards with suspicion the man or woman who has once been afflicted with insanity. It feels that he or she is not to be trusted; that there may be a recurrence of the mental disorder at the very moment when some particular and special bit of work has to be done. Many who have been insane and have recovered are certainly the victims of nervous instability; and while they may do much useful work if they be preserved from undue stress, they must to a certain extent live guarded lives, just as the phthisical individual has to avoid risks which another without his pulmonary weakness may incur with impunity. Here, again, adequate care and supervision of a modified character will do much either to prevent a recurrence of the mental disorder or at least to modify the character of the subsequent attack to such a degree that the symptoms do not necessitate the certification of the patient. It is very frequently difficult or even impossible for the friends or relations of the patient to give such assistance; their circumstances are so straitened that they cannot incur the responsibility; or, indeed, there may be no relatives to whom the patient can apply for assistance.

It is therefore obvious that there is ample scope for philanthropically disposed members of the community who are willing to give time and money to assist those who have recently recovered from a mental breakdown and whose circumstances are necessitous. Such work has been carried on for years past by the After-Care Association, the object of which is to help "poor persons discharged recovered from asylums for the insane." In the report of the Association for 1913 it is stated that 5,171 cases have been considered by the council since the work of the Association commenced. Some of these cases have been helped by judicious advice; for others assistance of a more material kind has been provided. "One of the most important functions of the Association," the report states, "is finding suitable occupation for persons who, although recovered, would probably have great difficulty in restarting themselves in life. The bestowal of a large amount of personal care and individual attention upon each case is necessitated, and the careful investigation of the suitability of those with whom they are placed. The result of this part of the work is far greater than the mere supplying of temporary homes, clothing, or grants for maintenance and tools, important though these may be."

Examples are given of the kind of work undertaken by the Association, and they serve to illustrate very clearly the utility of charitable effort in this direction. "A respectable married woman . . . was sent to a cottage home, an outfit given, and a situation found, where she did well and was able to support her youngest child." In another case, that of a "middle-aged widow," the patient was "boarded out in a country home and clothing given. Her old firm were seen and consented to re-engage her, and she is doing extremely well." These and similar cases, male and female, have been dealt with by the Association, and the benefits conferred upon a singularly deserving and necessitous class of the community are great.

There are, however, points in regard to the administration of the Association which are open to criticism. It does not seem, for instance, as if the right method has been adopted of getting into touch with those who might be benefited by the funds of the Association. There has evidently been a considerable accumulation of funds, so that the Association shows in its balance sheet for 1913 sums invested and on deposit amounting to over £6,000; whilst the excess of income over expenditure for the year ending December 31, 1913, was £1,418 16s. 2d.

Now the point is that these sums do not show necessarily that too much has been collected: it may possibly be more true to say that not enough has been expended, and this, not for the reason that there are not plenty of necessitous individuals requiring assistance, but because the Association has not been able to find them. The sums collected by the Association would probably be inadequate to deal with these. Possibly if some scheme of decentralisation were part of the policy of the Association more might be done in this way. For instance, certain sums might be placed at the disposal of the medical superintendents of the public asylums for them to disburse in the most fitting manner; or if it were considered inadvisable to delegate the responsibility to an individual, the moneys might be controlled by the committees of these institutions. Whoever might be appointed could be required to submit an account of disbursements to the central organisation. Patients discharged recovered from asylums might thus have all arrangements made for them by those with whom they had become more or less intimately acquainted; and in this way the necessity of recourse to a central authority, with all the additional time and trouble which such procedure involves, might be dispensed with. In such a scheme there is the possibility of the growth of an organisation which would extend its sphere of influence wider than does the present Association. It may be adduced in argument against this that already by the After-Care Association "cases are assisted from all parts of the country"; but it is practically certain—although the report does not appear to make this clear—that by far the largest numbers of cases come from the Metropolitan areas, as, *a priori*, one would be led to expect.

The Prisoners' Aid Society in its sphere certainly appears to have come nearer to the solution of the difficult problem of how to find out the necessitous cases, and then how to deal with them.

